# *QuickStats:* Percentage* of Currently Employed Adults Aged ≥18 Years Who Reported an Average of ≤6 Hours of Sleep^†^ per 24-Hour Period, by Employment Category^§^ — National Health Interview Survey, United States, 2008–2009 and 2017–2018^¶^

**DOI:** 10.15585/mmwr.mm6916a5

**Published:** 2020-04-24

**Figure:**
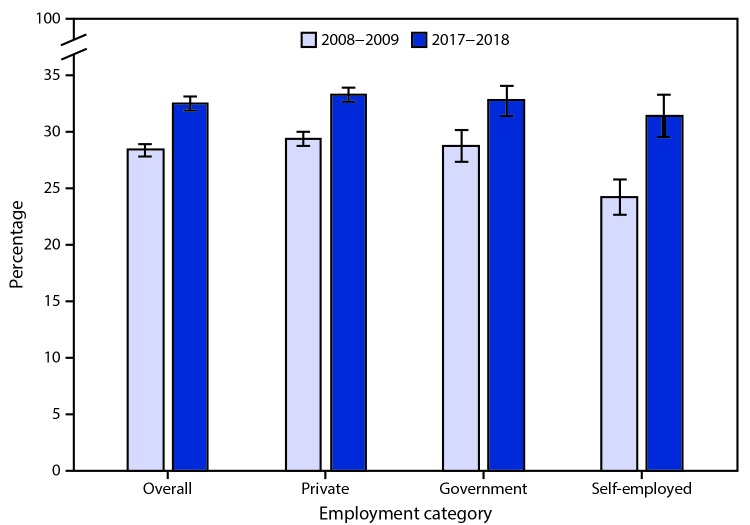
The percentage of employed adults who reported an average of ≤6 hours of sleep per 24-hour period increased from 28.4% during 2008–2009 to 32.6% during 2017–2018. During this period, increases were noted among private sector employees (29.5% to 33.3%), government employees (28.8% to 32.8%), and the self-employed (24.3% to 31.4%). A lower percentage of the self- employed reported ≤6 hours of sleep compared with private sector and government employees during 2008–2009. The smaller differences by employment categories noted during 2017–2018 were not statistically significant.

**Source:** National Health Interview Survey, 2008–2009 and 2017–2018. https://www.cdc.gov/nchs/nhis.htm.

